# Toxicity from Intra-Abdominal Injection of Chlorfenapyr

**DOI:** 10.1155/2013/425179

**Published:** 2013-04-16

**Authors:** Jina Lee, Jun Hyun Lee, Jong Min Baek, Do Sang Lee, Il Young Park, Jong Man Won, Ki Young Sung

**Affiliations:** Department of Surgery, Bucheon St. Mary's Hospital, The Catholic University of Korea, Sosa-dong, Wonmi-Gu, Bucheon City, Kyunggi-Do 420-717, Republic of Korea

## Abstract

*Introduction*. Chlorfenapyr is commonly used for food crops in Korea. However, chlorfenapyr toxicity in humans has not yet been studied. *Case*. A 74-year-old man was admitted to the emergency room after he intra-abdominally injected 20 mL of chlorfenapyr in an attempt to commit suicide. Emergency surgery was performed and accumulation of approximately 500 mL of reactive fluid in the abdomen was observed. The entire small intestine showed congestion. After surgery, additional surgery to drain the fluid was performed on POD 12. But immediately after administration of general anesthesia, flat rhythm was observed by electrocardiogram (ECG) monitoring, requiring cardiopulmonary resuscitation (CPR). *Discussion*. The color of the bowel was purple, indicating ischemic injury. This could be attributed to direct absorption of the substance through the peritoneum, leading to chemical injury to the small intestine serosa, unlike in the case of oral ingestion. This resulted in an ischemic change in the small intestine, eventually leading to sepsis. *Conclusion*. Only a few cases of chlorfenapyr toxicity have been reported in the literature, and death occurred in all cases, including our case. Therefore, careful and aggressive treatments are necessary. This is the first reported case of intra-abdominal injection of chlorfenapyr.

## 1. Introduction

Chlorfenapyr is a member of a new class of chemicals, namely, pyrroles (chemical name: 4-bromo-2-(4-chlorophenyl)-1-(ethoxymethyl)-5-(trifluoromethyl)-1H-pyrrole-3-carbonitrile; trade name: Pylon miticide-insecticide) [1]. Chlorfenapyr uncouples oxidative phosphorylation in the mitochondria, resulting in disrupted ATP production, cellular death, and ultimately, death of the organism. It is used for the removal of mites, caterpillar pests, thrips, and fungus gnats by foliar spray on ornamental crops in greenhouses. Chlorfenapyr is not recommended for outdoor use on cotton because of chemical persistence and concerns over its effects on avian reproduction. However, it remains in use for food crops in Korea. The compound is a light tan or light yellow solid powder, and it is usually dissolved in a solvent before use. While chlorfenapyr toxicity has not yet been studied in humans, studies in animals have led to the formulated product being classified as a toxicity category III chemical, indicating cautious use. In 2007, a case was reported in Japan in which a farmer who had inhaled chlorfenapyr vapors showed severe fatigue and died 7 days after exposure [2]. And in Korea, a 55-year-old man ingested chlorfenapyr and died after 7 days [3]. Here, we describe a case of a 74-year-old man who injected chlorfenapyr into his intra-abdominal space with suicidal intent.

Chlorfenapyr, which is a member of the pyrrole class of chemicals, is a proinsecticide compound; that is, its biological activity depends on its activation by another chemical. Oxidative removal of the *N*-ethoxymethyl group of chlorfenapyr by mixed-function oxidases forms the compound CL 303268. CL 303268 uncouples oxidative phosphorylation in mitochondria, resulting in disruption of ATP production, cellular death, and finally, mortality of the organism.

## 2. Case Presentation

**Figure 1:**
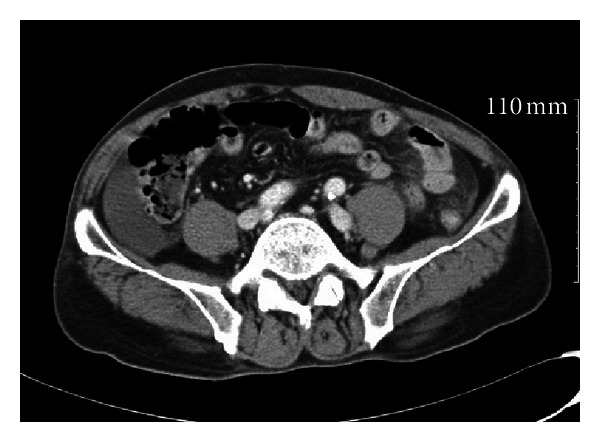
Abdominal computed tomography (CT) revealed fluid accumulation in the paracolic gutter on the right side.

**Figure 2:**
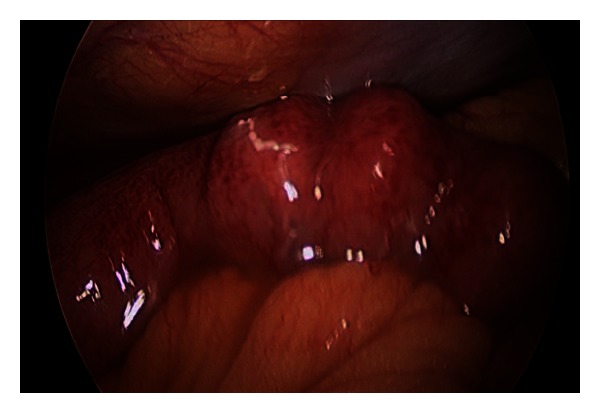
The entire small intestine appeared red and showed congestion.

A 74-year-old man was admitted to the emergency room with abdominal pain after he intra-abdominally injected 20 mL of chlorfenapyr by using a 10 mL syringe in an attempt to commit suicide. The patient had a history of idiopathic thrombocytosis, lower limb peripheral polyneuropathy, and major depressive disorder. The form of chlorfenapyr injected was an emulsion obtained by dissolving the chlorfenapyr in an aromatic solvent (Kocosol-100). The patient had undergone abdominal surgery 30 years ago for an air-pistol injury and had a transverse incision. The vital signs at the time of emergency room admission were as follows: blood pressure, 110/60 mmHg; heart rate, 79 beats/min; respiratory rate, 20/min; and body temperature, 36.2°C. The blood counts were as follows: white blood cell (WBC) count, 15,790 × 10^9^/L and platelet count, 258,000/L. No prolongation of prothrombin time was observed. The patient did not experience abdominal pain, but showed mild tenderness over the right abdomen. Abdominal computed tomography (CT) revealed fluid accumulation in the paracolic gutter on the right side (Figure 1). Emergency surgery was performed; accumulation of approximately 500 mL of reactive fluid in the abdomen was observed. Further, the fluid had a pesticide-like odor. The entire small intestine appeared red in color and showed congestion (Figure 2). Abdominal irrigation was performed, and surgery was completed after drain insertion. Third-generation cephalosporin and metronidazole were administered. After surgery, the patient was monitored in the intensive care unit. The patient did not have any abdominal pain, but his WBC count increased gradually. On postoperative day (POD) 6, he was transferred to the general ward and started on a liquid diet. The patient did not show symptoms of fever but showed continuous diaphoresis. Abdominal CT performed on POD 9 because of an increased WBC count revealed loculated fluid accumulation in the right upper abdominal area. Additional surgery to drain the fluid was performed on POD 12. Immediately after administration of general anesthetics, the patient's pCO_2_ level increased to 80 mmHg, and a flat rhythm was observed by electrocardiogram (ECG) monitoring, requiring cardiopulmonary resuscitation (CPR) to be performed in the operating room. After a normal sinus cardiac rhythm resumed, the patient was moved to the ICU with an abdominal drain. Arterial blood gas analysis (ABGA) performed under ventilator support in the ICU showed the following results: pH, 7.144; pCO_2_, 80.1 mmHg; pO_2_, 92.4 mmHg; HCO_3_^−^, 26.8 mmol/L; and O_2_ saturation, 94.0%. The patient showed signs of fever for the first time since admission and had a second cardiac arrest. The patient was administered CPR, but eventually died. Blood culture performed at the time of death was later found to be positive for multiple drug-resistant methicillin-resistant *Staphylococcus aureus* (MRSA).

## 3. Discussion

Chlorfenapyr is not a well-studied chemical; however, some studies have shown that the symptoms of chlorfenapyr toxicity include fever, diaphoresis, general fatigue, blurred vision, psychological effects, and rhabdomyolysis [2, 3]. We did not detect fever, nausea, vomiting, or psychological effects in our patient, but we did observe severe diaphoresis. However, we were unable to assess rhabdomyolysis. Unlike the findings reported previously, in this case, we found that the substance was injected intra-abdominally; the absorption route was assumed to be through the abdominal peritoneum. The amount injected was 20 mL, which was relatively lower than that observed in typical cases of oral ingestion. However, because the time between injection and surgery was about 8 h and the substance was likely to have been completely absorbed through the peritoneum, we believe that this relatively small dose led to the patient's symptoms and eventually his death.

During the surgery, the serosa of the small intestine showed an overall color change to red, indicating congestion, but no perforation or ischemic changes were observed; therefore, we performed irrigation. However, during the second surgery on POD 12, a severe abdominal adhesion was observed, and the color of the small and large intestines was purple, indicating ischemic injury. This could be attributed to direct absorption of the substance through the peritoneum, leading to chemical injury to the small intestine serosa, unlike in the case of oral ingestion, in which the substance is absorbed through the mucosa of the small intestine. We believe that this resulted in an ischemic change in the small intestine, eventually leading to sepsis. Other evidence of sepsis in this patient included fever, low blood pressure, and blood culture positive for MRSA. Third-generation antibiotics cephalosporin and metronidazole were administered according to the empirical observation of intra-abdominal inflammation. We regret that blood culture was not performed when ischemic injury of the bowel was suspected, although the patient did not have fever.

When the patient developed cardiac arrest during the second surgical procedure, we also observed severe hypercapnia. The cholinesterase level at the time of admission was a low value of 3921 U/L. The form of chlorfenapyr that the patient injected was dissolved in an aromatic organophosphate. Organophosphates suppress the activity of cholinesterases and lead to the stimulation of acetylcholine receptors. Symptoms can include fasciculation, cramps, weakness, twitching, paralysis, respiratory embarrassment, and cyanosis [4]. Respiratory acidosis is believed to be caused by myasthenia of the respiratory muscles and increased secretion from organ tissues [5]. During ventilator care, a decrease in the patient's pCO_2_ level was observed when the respiration rate had increased by 30. However, it was after the patient's death that we identified that chlorfenapyr was dissolved in an organophosphate solvent.

## 4. Conclusion

Only a few cases of chlorfenapyr toxicity have been reported in the literature, and death occurred in all these cases, including our case. Therefore, careful and aggressive treatments are necessary. This is the first reported case of intra-abdominal injection of this pyrrole derivative. Extensive irrigation was necessary during surgery. After surgery, hydration and hemodialysis were necessary during the early stages of treatment, similar to those in the cases of oral or vapor ingestion. If organophosphate toxicity is suspected, indicated by low cholinesterase levels, conservative treatments such as treatments with atropine and pralidoxime chloride are necessary. Considering the possibility of bowel ischemia, aggressive antibiotic treatment could also be required.
